# Transcriptomic Analysis Reveals Potential Gene Regulatory Networks Under Cold Stress of Loquat (*Eriobotrya japonica* Lindl.)

**DOI:** 10.3389/fpls.2022.944269

**Published:** 2022-07-22

**Authors:** Jiaying Zhang, Haishan An, Xueying Zhang, Fangjie Xu, Boqiang Zhou

**Affiliations:** ^1^Forestry and Pomology Research Institute, Shanghai Academy of Agricultural Sciences, Shanghai, China; ^2^Shanghai Key Laboratory of Horticultural Technology, Shanghai Academy of Agricultural Sciences, Shanghai, China

**Keywords:** *Eriobotrya japonica* Lindl., cold stress, physiological indicators, transcriptome analysis, differentially expressed genes

## Abstract

Loquat (*Eriobotrya japonica* Lindl. ) is one of the most economically important evergreen fruit crops in China, while it often suffered the injury of cold stress in winter and earlier spring, and the annual yield loss of loquat fruits caused by cold or freezing stress was immeasurable. However, knowledge about the physiological response and molecular mechanism under cold stress is still limited. To investigate the potential regulation mechanism pre- and post-cold stress in loquat and the changes in physiological indicators, a comparative transcriptome analysis was performed against a cold-resistant cv. “Huoju” and a cold-sensitive cv. “Ninghaibai”. The results of physiological indicators related to cold resistance indicated that rachis was most sensitive to cold stress and was considered as the representative organ to directly evaluate cold resistance of loquat based on subordinate function analysis. Here, we compared the transcriptome profiles of rachis pre- and under cold stress in “Huoju” and “Ninghaibai”. A total of 4,347 and 3,513 differentially expressed genes (DEGs) were detected in “Ninghaibai” and “Huoju”, among which 223 and 166 were newly identified genes, respectively, most of them were functionally enriched in plant hormone signal transduction (Huoju: 142; Ninghaibai: 200), and there were higher plant hormone content and related DEG expression levels in “Huoju” than that of “Ninghaibai”. Moreover, a total of 3,309 differentially expressed transcription factors (DETFs) were identified, and some DEGs and DETFs were screened to be subjected to co-expression network analysis based on the gene expression profile data. Some candidate DEGs, including UDP-glycosyltransferase (UGT), glycosyltransferase (GT), sugar phosphate/phosphate translocator (SPT), sugar transport protein (STP), proline-rich receptor-like protein kinase (PERK), and peroxidise (POD), were significantly affected by cold stress, and the expression level of these genes obtained from real-time quantitative RT-PCR was consistent with the pattern of transcriptome profile, which suggested that these genes might play the vital roles in cold resistance of loquat. Our results provide an invaluable resource for the identification of specific genes and TFs and help to clarify gene transcription during the cold stress response of loquat.

## Introduction

Loquat (*Eriobotrya japonica* Lindl. Rosaceae, Maloideae), which is originated from China, is a typical subtropical, evergreen, fruit-bearing tree (Lou et al., 2018; Wu et al., 2018b). Loquat is sweet and sour, with thin and juicy skin, rich in nutrient, such as glucose, fructose, multiple vitamins, organic acids, and mineral ions, and wide consumption in China (Yan et al., 2021). But the phonological characteristics of loquat are disparate distinctively from those of other Rosaceae family trees; it blooms generally in autumn, and the young fruit development occurs in winter and early spring, during which the ambient temperature was the lowest of the whole year in China; therefore, cold stress or freezing injury was the main limiting factor that affects loquat yield and fruit quality (Lou et al., 2018; Li et al., 2020; Pan et al., 2020). According to the statistics, the annual loquat yield and economic loss caused by extreme cold weather were 13,300 and 65 million RMB, respectively. In the past decades, the breeding of loquat was gradually inclined to cold-resistant resource selection, and a series of novel varieties or superiors with enhanced cold-resistant trait were successfully realized (Lou et al., 2018). Consequently, enhancement in cold resistance was becoming an important area for agricultural investigation and crop improvement. Therefore, it is exigent to explore the molecular mechanism underlying cold stress response and tolerance in loquat and identify the key genes that could be using for the breeding of novel loquat varieties with enhanced cold resistance to ensure yield under such unfavorable conditions. Regrettably, up to date, the molecular mechanism regulating cold-responding cues in loquat has not been fully elucidated, so it was urgent to reveal the related regulatory pathways which would not only facilitate the molecular-assisted selection conferring cold resistance to loquat, but also help to stabilize the production and improve the quality of loquat.

Cold stress is devastating abiotic stress that significantly affects plant growth and development, reduces quality and productivity, and even limits their geographical distribution (Cao and Zheng, 2018; Qiang et al., 2018). Over a long evolutionary period, plants have developed a series of adaptive mechanisms to survive under terrible conditions (Wen et al., 2018; Ji et al., 2020; Qi et al., 2020; Li et al., 2021; Zhang et al., 2022). To respond and adapt to cold stress, an array of physiological alterations was induced to enable them to grow normally with an enhanced cold tolerance (Liu et al., 2018; Li et al., 2019). In addition, numerous cold-associated genes or transcription factors (TFs) were triggered, which thereby leads to certain signal transduction pathways to resist cold stress at the cell and whole-plant levels (Wu and Zhang, 2020). During the course of cold stress, some biochemical components and physiological indicators, such as proline, malondialdehyde (MDA), soluble sugar, reactive oxygen species (ROS), superoxide dismutase (SOD), peroxidase (POD), and catalase (CAT), may be up or downregulated in plants (Valitova et al., 2019; Sqwab et al., 2020; Wang et al., 2020). The degree of change in these indicators could reflect the cold resistance of plants. Some genotypes with higher amounts of proline, soluble sugar, SOD, POD, and CAT were designated tolerant to cold. All these changes have been associated with the regulation of gene transcription. To respond to cold stress, functional genes and TFs are induced to directly protect cells and modulate gene expression for signal transmission (Hou et al., 2019; Dong et al., 2021). The ICE-CBF-COR pathway is suggested to be the most important mechanism and a well-known pathway for cold stress resistance in plants (Zhang et al., 2011). In addition, TFs are the master regulators governing the expression of many target genes in a network mode of a single TF of downstream genes, in which APETALA2/ethylene-responsive element-binding factor (AP2/ERF), basic helix-loop-helix (bHLH), MYB, NAC, WRKY, DRE-binding protein (DREB), and basic domain leucine zipper (bZIP) were suggested to play the vital roles in modulating cold stress-responsive gene expression in plants (Xing et al., 2019; Xu et al., 2019). In the recent years, even though an increasing number of cold-induced genes and TFs were isolated and identified sequentially in Rosaceae fruit trees, including pear (Dong et al., 2021), apple (Xu et al., 2018b), loquat (Hong et al., 2018; Xu et al., 2018a), rice (Rabbani et al., 2003), maize (Hayano et al., 2009), and so on, the expression patterns and regulatory mechanism of genes or TFs related to cold stress response were yet to be clearly defined in these fruit trees, especially in loquat.

RNA-sequencing technology is a powerful method of identify the novel transcripts and analyzing gene expression profiling (Wu and Zhang, 2020; Raza et al., 2021). It enables rapid gene discovery and creates more sensitive and accurate transcriptome profiles than other techniques. Recently, the researchers have used RNA-seq technology to analyze the transcriptome regarding specific biological traits in loquat, and a great deal of candidate genes or transcripts has been successfully explored, such as flower bud differentiation (An et al., 2021), fruit development and ripening (Song et al., 2016), and fruit lignification during postharvest cold storage (Liu et al., 2019). However, the detail transcription information, as well as the genetic regulatory networks of loquat under clod stress conditions was open, and the identification of key genes or regulators that respond to cold stress by RNA-seq is very necessary.

The object of this present work was to evaluate physiological indicators, including proline, soluble sugar, MDA, ROS (H_2_O_2_ and ${\text{O}}_{2}^{-}$), SOD, POD, and CAT, associated with resistance to cold stress in different organs using two loquat varieties with distinct differences in cold resistance, one was “Huoju” (cold-resistant) and another one was “Ninghaibai” (cold-sensitive). Based on the subordinate function analysis, rachis was screened as a representative organ that directly evaluated the cold resistance of loquat. Additionally, a comparative transcriptome analysis was carried out to explore novel genes or transcription factors regulating cold tolerance in loquat. The results of this study would not only provide new insights into discovering the gene expression patterns and signaling pathways involved in responding of cold resistance and offer a theoretical foundation and technical assistance for cold resistance breeding of loquat.

## Materials and Methods

### Plant Materials

**Figure 1:**
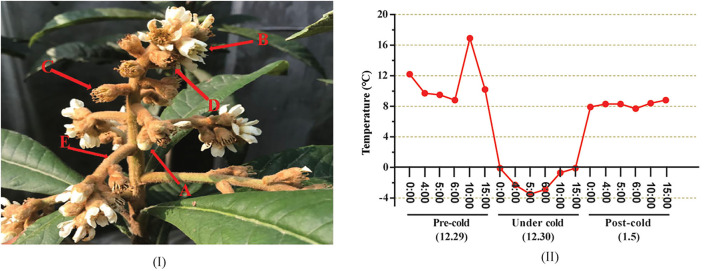
Schematic diagram of different organs of loquat inflorescence and dynamics changes of temperature (°C) at different time points of samples collected. (A–D) indicates the different developmental stages of floral organ (A, showing-white flower; B, blooming flower; C, unclosed calyx; D, closed calyx). (E) indicates the rachis of inflorescence; The temperatures during the period of 0:00–15:00 on 29 December (pre-cold) were all above 8°C and the maximum temperature reached 17°C (10:00 am); the temperatures throughout the day on 30 December (under cold) were all below 0°C, the lowest temperature drop to −3.5°C (5:00 am); the average temperature during the period of 0:00–15:00 on 5 January (post-cold) invariably maintained at 8°C.

A number of two loquat (*Eriobotrya japonica* Lindl.) varieties with different cold resistances were used in this study. “Huoju” is a cold-resistant genotype, whereas “Ninghaibai” is a cold-sensitive cultivar (Zhang et al., 2021b). Both were planted at the Jinshan Fruit Tree Experimental Station of Shanghai Academy of Agricultural Sciences, Shanghai, China (30°47′27″; 121°8′6″). Floral organs at different developmental stages, including showing-white flowers, blooming flowers, unclosed calyxes, closed calyxes, and rachises (Figure 1A), were sampled, respectively, at 10:00 a.m. on 29 December 2020 (pre-cold stress, designated H29 and N29), 6:00 a.m. on 30 December 2020 (under cold stress, designated H30 and N30), and 10:30 a.m. on 5 January 2021 (post-cold stress, designated H5 and N5), and the temperature changes over these 3 days were accordingly recorded and showed minutely in Figure 1B. Fresh samples were stored for the determination of ROS (H_2_O_2_ and ${\text{O}}_{2}^{-}$), plant hormones, and antioxidant enzymes activities, including POD, SOD, and CAT. Other samples were immediately frozen in liquid nitrogen and stored at −80°C for further analysis of soluble sugar, proline, MDA, and transcriptome profiles with three biological replications.

### Cold Treatment

A number of 10 inflorescences (as shown in Figure 1A) were randomly collected from “Huoju” and “Ninghaibai”, respectively, and were brought back to the laboratory. Subsequently, all the inflorescences were cultured in bottles filled with water and moved into a growth chamber. The treatment temperature was set at 4°C, and the rachises of inflorescences were sampled at 0, 1, 5, 10, 15, 24, 48, and 72 h after cold treatment. Samples collected at each indicated time point were immediately frozen in liquid nitrogen and stored at −80°C for qRT-PCR analysis with three biological replications.

### Physiological Indicator Determination

The content of soluble sugar and MDA was determined according to the method described by Huang et al. (2010). About 0.5 g samples were homogenized with an equal volume of 0.5% (w/v) 2-thiobarbituric acid in 20% (w/v) trichloroacetic acid. The homogenate was incubated at 95°C for 30 min and then centrifuged at 16,000 g for 30 min. The supernatant was used for MDA and soluble sugar determination; the measurement of proline content was carried out by referring to the experimental method of “Plant Physiological Experiment.” Then, 0.5 g samples were homogenized in 500 μl of water. Following centrifugation, 200 μl of the homogenate was added to 200 μl glacial acetic acid and 200 μl of ninhydrin solution in a capped tube. The solutions were mixed and incubated for 100 min at 100°C. Following incubation, the samples were extracted with an equal volume of xylene, and the absorbance of the aqueous phase was quantified at 522 nm in reference to a proline standard curve (Merwad et al., 2018); ROS (H_2_O_2_, ${\text{O}}_{2}^{-}$) and antioxidant enzyme (SOD, POD, and CAT) activity were quantified using the relevant detection kits (Nanjing Jiancheng Bioengineering Institute) based on the manufacturer's instructions. The contents of four major endogenous plant hormones, i.e., auxin (IAA), abscisic acid (ABA), gibberellin 3 (GA3), and zeatin were determined based on the method of liquid chromatography-mass spectrometry (LC-MS) described by An et al. (2021). About 0.5 g of fresh tissues from each sampled time was grinded in liquid nitrogen and was digested in 5 ml ethyl acetate for 12 h at 4°C, the supernatant was dried by nitrogen flow at 25°C, then dissolved by 300 μl chromatographic methanol and then ultrasonically extracted for 10 min. Finally, the solution was filtered with the 0.22-μm membrane filters and 5 μl was injected for analysis.

### Total RNA Extraction and RNA-Seq

Total RNAs were extracted from loquat rachises using the Polysaccharides and Polyphenolics-rich RNAprep Pure Plant Kit (TIANGEN, China) according to the manufacturer's protocol. RNA integrity was validated with an Agilent 2100 Bioanalyzer (Agilent Technologies, Santa Clara, CA, USA). RNA-seq libraries were constructed with a TruSeq Stranded mRNA LTSample Prep Kit (Illumina, San Diego, CA, USA) and applied to an Illumina Nova Seq 6000 sequencing system guide for RNA-seq analysis by Biomarker Technologies Corporation (Beijing, China).

### Functional Annotation

Gene functions were annotated by aligning the genes with the nonredundant (NR) and nucleotide (NT) sequences of the National Center for Biotechnology Information (NCBI), Swiss-Prot, Kyoto Encyclopedia of Genes and Genomes (KEGG), Clusters of Orthologous Groups of proteins (COGs), and Gene Ontology (GO) databases. For this purpose, Basic Local Alignment Search Tool (BLASTx) for searching protein databases using a translated nucleotide query was used for gene annotation at a threshold *E*-value of 10^−5^. Proteins with the highest numbers of hits to the genes were used to assign functional annotations to them. Gene Ontology classification was performed by mapping the relationship between SwissProt and the GO terms. The genes were mapped to the Kyoto Encyclopedia of Genes and Genomes database to annotate their potential metabolic pathways (Kanehisa et al., 2008). The gene sequences were also aligned to the Cluster of Orthologous Groups database to predict their function.

### Identification and Phylogenetic Analysis of Differentially Expressed Genes Response to Cold Stress

To calculate the expression of genes, the fragments per kilobase per million mapped reads (FPKM) and read count values for each gene were calculated with Bowtie2 and eXpress (Trapnell et al., 2010; Langmead and Salzberg, 2012; Robert and Pachter, 2013). This method can eliminate the influence of gene length on the calculations of gene expression, and the results can be directly used to compare different gene expression patterns among samples. Differentially expressed genes (DEGs) were identified with the DESeq functions size factors and nbinom test (Anders and Huber, 2013). *p-*value < 0.05 and fold change > 2 were set as the thresholds for significant differential expression. A hierarchical cluster analysis of the DEGs was performed to explore transcript expression patterns. GO and KEGG pathway enrichment analyses of the DEGs were performed on the hypergeometric distribution. In addition, sequence alignment using proteins, which were encoded by these DEGs, was conducted and a phylogenetic tree was constructed by the maximum likelihood (ML) method using MEGA software (version 6.0). A bootstrap of 1,000 replicates with pairwise deletion option was performed.

### Expression Validation of Genes With Real-Time Quantitative RT-PCR

Total RNAs were extracted from the rachises of two loquat varieties collected at 4°C at the indicated time points. For qRT-PCR analysis, first-strand cDNA synthesis was performed using the SYBR Prime Script RNA RT–PCR Kit (TaKaRa, Japan). qRT-PCR was performed using the Light Cycler 480 (Roche, USA). qRT-PCR was carried out with the SYBR-Green PCR kit (TaKaRa, Japan) using 20 μl of reaction mixture consisting of 10 μl of 2 × SYBR-PreMix EX Taq, 50 ng of cDNA, and 0.25 μm of each primer. The qPCR program was set as follows: denaturation at 94°C for 5 min, followed by 40 cycles of 94°C for 10 s, 60°C for 30 s, 72°C for 30 s, and a final extension for 3 min at 72°C. All gene-specific primers from the identified genes for real-time PCR were designed using SnapGene software (Supplementary Table S1). All qRT-PCRs were normalized using Ct value corresponding to the *EjACTIN* gene (as the internal reference) with access number AB710173 (Song et al., 2017; Lou et al., 2018). Template-free controls were included in each run for each primer pair. Relative expression levels of each gene were calculated using the 2^−Δ*ΔCp*^ algorithm (Zhang et al., 2021a). Each gene has three biological repeats and three technical repeats.

### Data Analysis

The data were subjected to one-way analysis of variance (ANOVA) in SPSS v. 18.0 (SPSS Inc., Chicago, IL, USA). A significant difference was considered at the level of *p* < 0.05 by Duncan's test. The results were shown as mean ± standard error (SE) (*n* = 3), and the graphs were created by GraphPad Prism 8.0 (GraphPad Software Inc., La Jolla, CA, USA).

## Results

### Changes in Soluble Sugar, Proline, and MDA in Loquat Under Cold Stress

**Figure 2:**
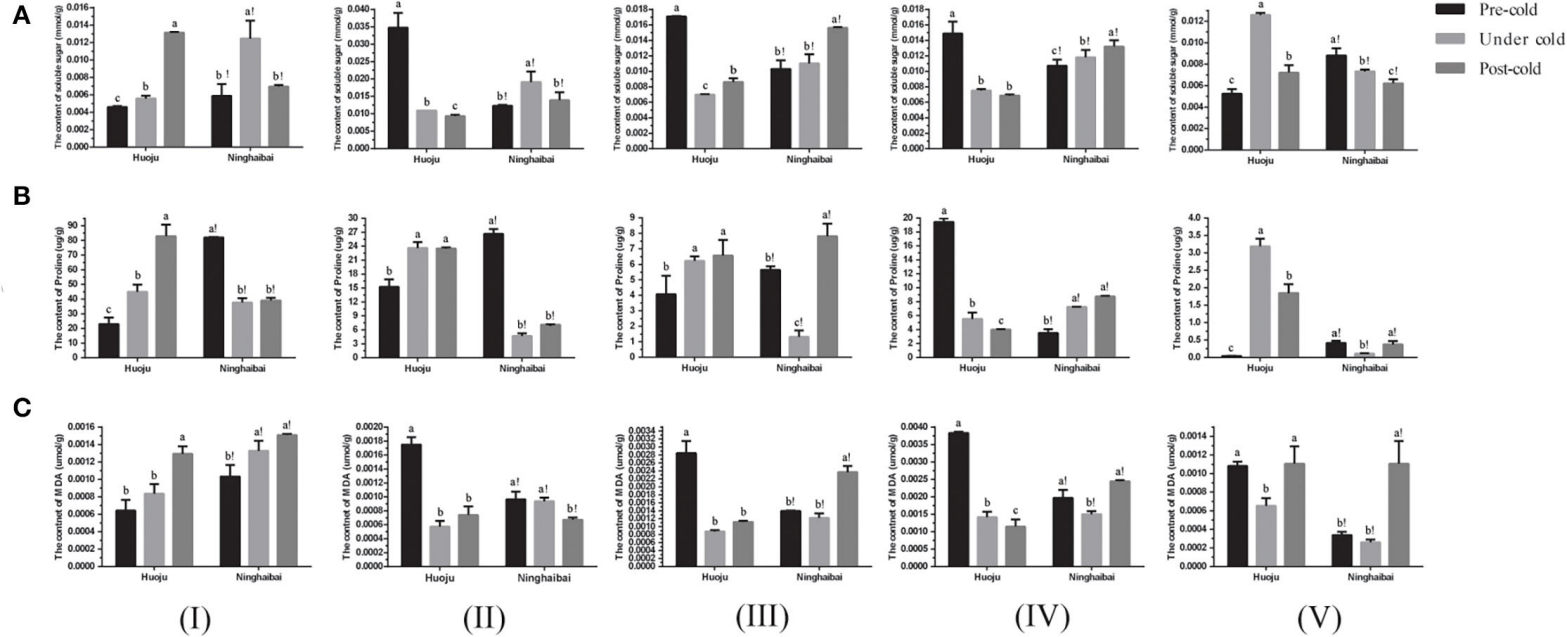
Changes in content of soluble sugar **(A)**, proline **(B)**, and MDA **(C)** in different organs of two loquat varieties. I–V in the graph indicates sampling organs, including showing-white flower, blooming flower, unclosed calyx, closed calyx, and rachis. Different letters indicate the significance at *p* < 0.05, out of which a, b, and c indicate the significant difference of “Huoju,” whereas a!, b!, and c! indicate the significant difference of “Ninghaibai.” Error bars indicate the standard deviation based on three replicates.

It indicated that contents of soluble sugar, proline, and MDA varied significantly during the period of pre-, under, and post-cold stress (Figure 2). As for “Huoju”, the content of soluble sugar increased obviously in organs of showing-white flower and rachis specially that of rachis under cold stress, but it declined sharply in organs of the blooming flower, unclosed calyx, and closed calyx (Figure 2A); as for “Ninghaibai”, the soluble sugar content showed a “down-up-down” trend both in the showing-white and in the blooming flowers, and a continuous increment in unclosed calyx and closed calyx, but it decreased gradually in rachis (Figure 2A).

The content of proline continued to the increase in showing-white flowers, blooming flowers, and unclosed calyxes of “Huoju”, whereas the proline content decreased first and then increased under cold stress in the same organs of “Ninghaibai”. The proline content was decreased in the closed calyx of “Huoju” but increased in that of “Ninghaibai”. There was a reverse trend in the proline content of rachises between “Huoju” and “Ninghaibai”, and the proline content first increased and then decreased in “Huoju” but first declined and then increased in “Ninghaibai” after cold treatment (Figure 2B).

The content of MDA increased continuously both in showing-white flowers of “Huoju” and “Ninghaibai”. The MDA content decreased sharply and then rose slightly in blooming flowers, unclosed calyxes, and rachises of “Huoju”, but it displayed a continuous decrement only in blooming flowers of “Ninghaibai”. The changes in MDA content in unclosed calyxes, closed calyxes, and rachises of “Ninghaibai” exhibited a similar tendency, and it decreased initially and then increased (Figure 2C). In total as mentioned above, the contents of soluble sugar and proline in the rachis of cold-resistant cv. “Huoju” were clearly higher than those of the corresponding organs in cold-sensitive cv. “Ninghaibai”, suggesting that soluble sugar or proline played the important roles in responding cold stress and could be considered as the indicator associated with cold resistance. On the whole, the dynamic trend of content of physiological indicators in rachis at different time points under cold stress could accurately indicate the role of soluble sugar, proline, and MDA in enhancing loquat cold resistance. The increase in soluble sugar and proline content and the decrease in MDA content in rachis could protect loquat against cold injury.

### Changes in POD, SOD, CAT, H_2_O_2_, and ${\text{O}}_{2}^{-}$ in Loquat Under Cold Stress

**Figure 3:**
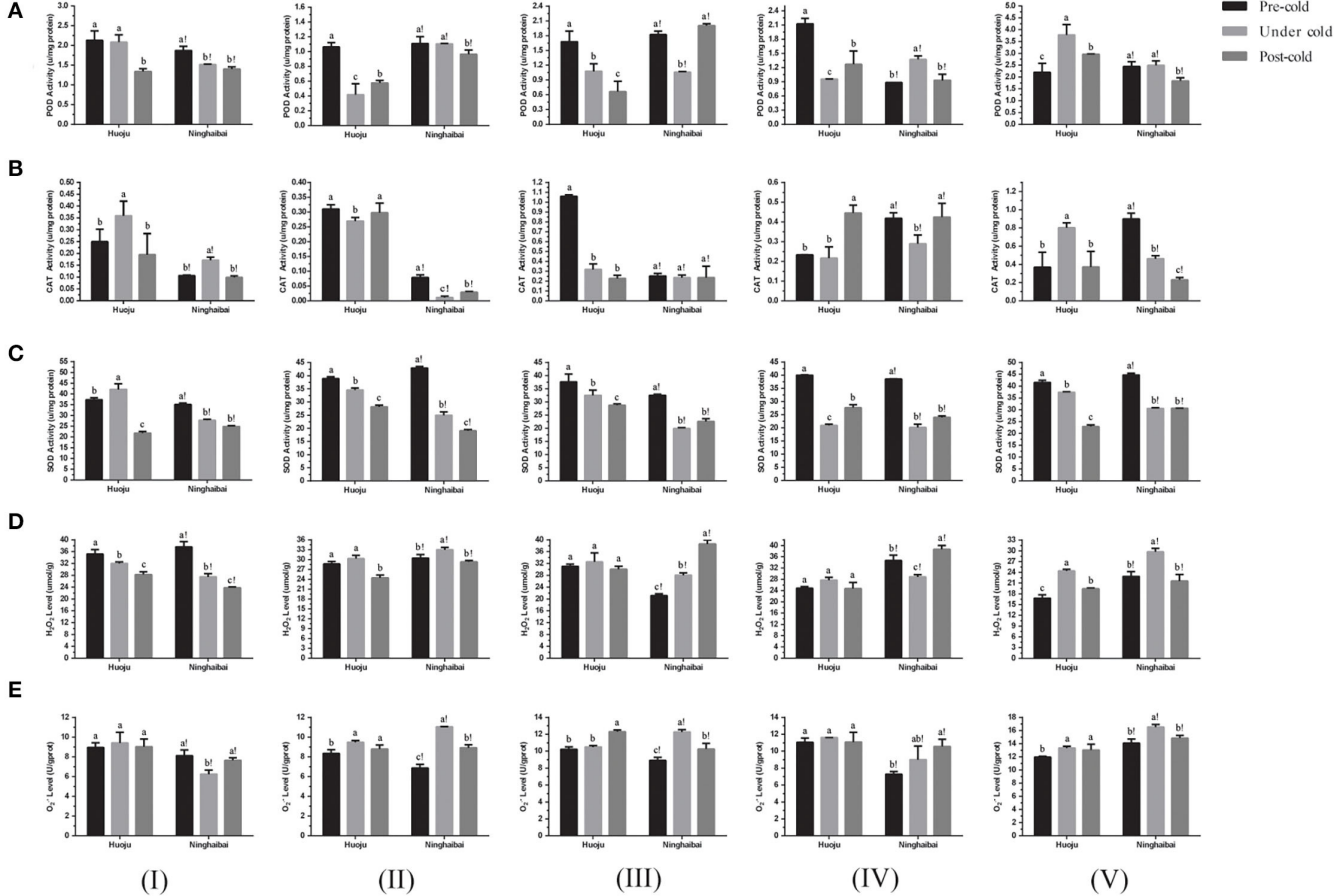
Changes in activity of antioxidant enzymes POD **(A)**, CAT **(B)**, and SOD **(C)** and level of H_2_O_2_ **(D)** and ${\text{O}}_{2}^{-}$ **(E)** in different organs of two loquat varieties. I–V in the graph indicates sampling organs, including showing-white flower, blooming flower, unclosed calyx, closed calyx, and rachis. Different letters indicate the significance at *p* < 0.05, out of which a, b, and c indicate the significant difference of “Huoju,” whereas a!, b!, and c! indicate the significant difference of “Ninghaibai.” Error bars indicate the standard deviation based on three replicates.

The POD activity decreased in showing-white flowers, blooming flowers, unclosed calyxes, and closed calyxes but increased obviously in rachises of “Huoju” under cold stress. In “Ninghaibai”, the POD activity increased obviously in unclosed calyxes under cold stress, but decreased in other four organs especially in closed calyxes under cold stress (Figure 3A). The showing-white flowers, blooming flowers, and unclosed calyxes of “Huoju” possessed higher CAT activity than their corresponding organs in “Ninghaibai” under cold stress. In the rachis, the CAT activity was higher in “Ninghaibai” than in “Huoju” in the period of pre-cold stress, whereas it was decreased sharply in “Ninghaibai” and increased strongly in “Huoju” under cold stress, and the CAT activity of “Ninghaibai” surpassed that of “Huoju” (Figure 3B). There was a consistent dynamic trend of SOD activity in the same organs in both “Huoju” and “Ninghaibai”. The SOD activities of all organs in “Huoju” were stronger than those in “Ninghaibai” under cold stress (Figure 3C). The H_2_O_2_ level decreased in showing-white flowers while increased in blooming flowers, unclosed calyxes, closed calyxes, and rachises of “Huoju” under cold stress. In the showing-white flowers and closed calyxes of “Ninghaibai”, the H_2_O_2_ level significantly decreased under cold stress. In the other tested organs of “Ninghaibai”, the H_2_O_2_ level presented an increasing trend under cold stress (Figure 3D). The ${\text{O}}_{2}^{.-}$ level increased in all tested organs of “Huoju” under cold stress. In “Ninghaibai”, except for showing-white flowers, the ${\text{O}}_{2}^{-}$ level also increased in other tested organs under cold stress (Figure 3E). In the whole, cold stress induced the increase in ROS (H_2_O_2_ and ${\text{O}}_{2}^{-}$) level in most tested organs both in “Huoju” and in “Ninghaibai”, and the antioxidant enzyme activities were higher in “Huoju” than that in “Ninghaibai”, especially in rachises, under cold stress. Thus, the effect of scavenging ROS was stronger in “Huoju” compared with “Ninghaibai”, resulting in the lower ROS level in “Huoju” under cold stress, and this phenomenon was easier to observe in rachises.

### Membership Degree Analysis of Respond to Cold Stress of Different Organs in Two Loquat Varieties

To screen the representative organ that could directly evaluate cold resistance of loquat *via* determining physiological indicators associated with cold resistance, the membership degree of respond to cold stress of different organs was analyzed and the results are minutely shown in Table 1. In both two loquat varieties, the membership of rachis was highest than other four tested organs. The membership of blooming flower was second-largest in “Huoju”, followed by closed calyx, showing-white flower, and unclosed calyx. Nevertheless, the membership of blooming flower was lowest than other organs, and the membership sort of different organs was rachis > closed calyx > unclosed calyx > showing-white flower > blooming flower in “Ninghaibai”. Meanwhile, the above physiological indicators, including soluble sugar, proline, POD, CAT, and ROS, most significantly changed in rachises than other tested organs, and the dynamic trend of physiological indexes in rachises easily indicated the difference in cold resistance in two loquat varieties. These results showed that rachis was closely related to cold resistance and could be as a representative organ to directly assess loquat cold tolerance under cold stress.

**Table 1:** Membership degree of response to cold stress of different organs of “Huoju” and “Ninghaibai”.

	**Soluble sugar**	**MDA**	**Proline**	**POD**	**SOD**	**CAT**	**H_**2**_O_**2**_**	** ${\text{O}}_{2}^{-}$ **	**Average membership**	**Order**
**Huoju**										
Showing-white flower	0.374	0.462	0.444	0.545	0.388	0.503	0.489	0.371	0.447	4
Blooming flower	0.317	0.397	0.636	0.488	0.530	0.475	0.554	0.508	0.488	2
Unclosed calyx	0.388	0.327	0.538	0.385	0.382	0.302	0.322	0.323	0.377	5
Closed calyx	0.329	0.410	0.358	0.479	0.459	0.377	0.531	0.678	0.453	3
Rachis	0.440	0.509	0.491	0.483	0.579	0.471	0.548	0.527	0.506	1
**Ninghaibai**										
Showing-white flower	0.392	0.624	0.382	0.393	0.436	0.367	0.380	0.573	0.443	4
Blooming flower	0.355	0.487	0.365	0.529	0.417	0.387	0.398	0.538	0.435	5
Unclosed calyx	0.443	0.392	0.538	0.401	0.403	0.487	0.452	0.443	0.445	3
Closed calyx	0.488	0.524	0.683	0.582	0.440	0.512	0.494	0.426	0.519	2
Rachis	0.447	0.589	0.529	0.570	0.666	0.482	0.496	0.478	0.532	1

### Sequence Analysis and Assembly

To obtain a better understanding of the molecular genetic mechanisms underlying the cold tolerance of loquat varieties under low-temperature conditions, a comparative transcriptomic analysis was performed *via* RNA-seq. A total of four samples collected at time point of pre-cold (H29 and N29) and under cold (H30 and N30) with three biological replicates were subjected to RNA-seq analysis, and a total of 12 RNA-seq libraries were constructed and sequenced using the Illumina sequencing platform. Through rigorous quality estimation and data cleaning, 21.69–24.16 M clean reads with Q30 bases (those with a base quality >30) remained as high-quality reads for further analysis. Their Q30 and GC content ranges were 91.36–91.62% and 47.31–48.01%, respectively. Moreover, the *N* percentage was 0.00% in all samples (Table 2).

**Table 2:** Summary of Illumina transcriptome sequencing for loquat rachis under cold stress.

**Cultivar**	**Sampling data**	**Clean reads**	**Q30 (%)**	**GC (%)**	***N*** **percentage (%)**
“Huoju”	H29	21,686,773	91.62	47.51	0.00
	H30	23,033,429	91.42	47.61	0.00
“Ninghaibai”	N29	24,158,257	91.36	47.31	0.00
	N30	22,314,566	91.55	48.01	0.00

### Identification of DEGs Responded to Cold Stress in Loquat

The DEGs between samples from cold stress treatment were identified by pair-wise comparisons of two cDNA libraries (H29-vs.-H30, N29-vs.-N30), and the FPKMs of all DEGs were calculated. The differences in gene expression were evaluated based on *p* < 0.05 and fold change > 2, and DEGs obtained by RNA-seq were matched to gene IDs in the loquat genomic database. A total of 7,860 genes were significantly differentially expressed between these two libraries. Of these genes, 2,677 genes were upregulated and 5,183 genes were downregulated (Table 3). A total of 3,513 DEGs were detected for the H29-vs.-H30 library, with 1,105 upregulated and 2,408 downregulated; 4,347 DEGs (1,572 upregulated and 2,775 downregulated) were detected for the N29-vs.-N30 library (Table 3; Supplementary Figures S1a,b). The comparative profile of DEGs in both libraries showed that more DEGs were downregulated than upregulated genes. GO and KEGG enrichment analyses revealed 666 upregulated and 1,852 downregulated transcripts common to both libraries (Supplementary Figures S1c,d).

**Table 3:** Statistics of upregulated or downregulated genes in each library.

**Item**	**Upregulated**	**Downregulated**	**Total**
H29-vs.-H30	1,105	2,408	3,513
N29-vs.-N30	1,572	2,775	4,347
Total	2,677	5,183	7,860

### Functional Classification and Annotation of DEGs

**Figure 4:**
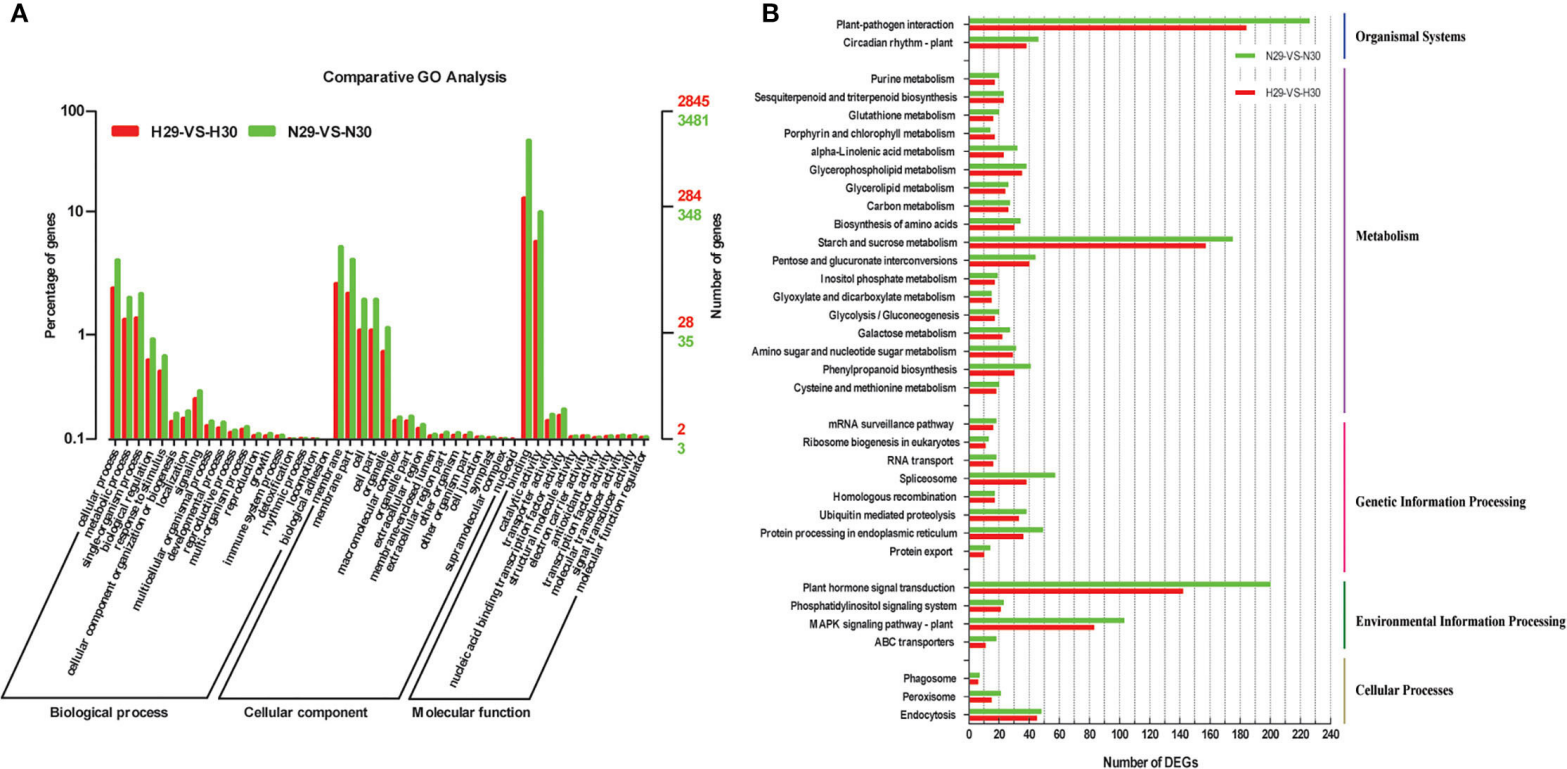
Functional annotation of loquat transcripts based on gene ontology (GO) categorization **(A)** and KEGG classification **(B)** of DEGs. I, a total of 25,655 DEGs were classified into three main categories, including “biological process,” “cellular component,” and “molecular function.” The left y-axis represented the percentage of a specific category of DEGs in the main category. The right y-axis represented the number of DEGs in each category; II, a total of 2,820 DEGs were classified into five main categories, including organismal systems, metabolism, genetic information processing, environmental information processing, and cellular processes.

The functions of predicted DEGs were classified with GO, COG, and KEGG assignments. A total of 25,655 DEGs were enriched in GO terms, out of which 11,472 and 14,183 DEGs were identified in “Huoju” “Ninghaibai”, respectively, and these DEGs were categorized into three kinds: biological process, cellular component, and molecular function. The number of DEGs in each GO term of “Ninghaibai” was higher than that of “Huoju”. Among all, 19 and 21 GO terms were significantly involved in the biological process, cellular component, and molecular function categories of “Huoju” and “Ninghaibai”, respectively (Figure 4A; Supplementary Table S2). Under the biological process category, five GO terms were enriched in cellular process, metabolic process, single-organism process, biological regulation, and response to stimulus in both varieties. In the cellular component domain, membrane part and cell part were the most highly represented terms, followed by organelle and macromolecular complex terms. As for the molecular function category, a most of DEGs were categorized under binding, catalytic activity, transporter activity, and nucleic acid-binding transcription factor activity terms. These results demonstrated that DEGs involved biological regulation, response to stimulus, binding, catalytic activity, and transporter activity might play important roles in the responding of cold stress in loquat.

To further predict and classify the DEGs, 2,932 DEGs were totally annotated by searching against the COG dataset. COG-annotated putative DEGs were functionally classified into at least 26 categories, such as cellular structure, biochemistry metabolism, molecular processing, and signal transduction, and the number of DEGs in all COG categories of “Ninghaibai” was higher than that of “Huoju” (Supplementary Figure S2, Supplementary Table S3). The cluster for signal transduction mechanisms represented the largest group, followed by general function prediction, carbohydrate transport and metabolism, and cell wall/membrane/envelope biogenesis, and cytoskeleton, extracellular structures, and RNA processing and modification represented the smallest groups in both loquat varieties.

All DEGs were mapped to reference canonical pathways in the KEGG database, which is an alternative approach to categorize gene functions with an emphasis on biological pathways. A total of 2,820 DEGs (Huoju: 1,278, Ninghaibai: 1,542) with annotated KEGG results were classified into five main categories, including organismal systems, metabolism, genetic information processing, environmental information processing, and cellular processes, based on the type of KEGG pathways. As shown in Figure 4B, the DEGs were mainly concentrated upon plant hormone signal transduction, starch and sucrose metabolism, plant–pathogen interaction, and MAPK signaling pathway plant (Supplementary Table S4) and were selected for subsequent analysis. The number of DEGs in all KEGG pathway categories was higher in “Ninghaibai” than in “Huoju”, and the results were similar to those of GO and COG analyses.

### DEGs Related to the Plant Hormone Signaling Transduction Pathway and Changes in Plant Hormone Content in Rachises Under Cold Stress

**Figure 5:**
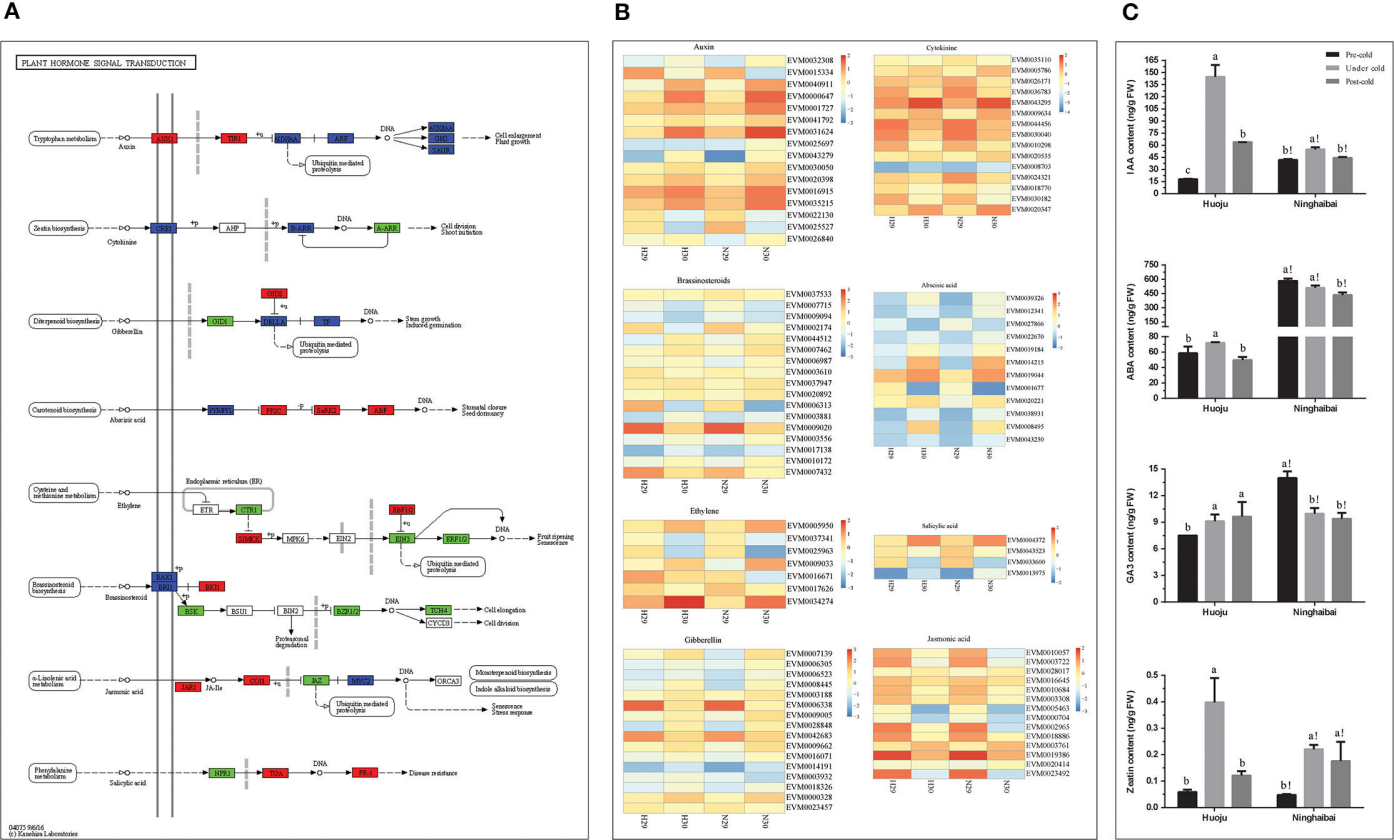
The KEGG pathway annotation diagram of the “plant hormone signal transduction pathway” of DEGs and dynamic changes of endogenous hormones in loquat rachises under cold stress. **(A)** KEGG analysis exhibiting the enrichment of DEGs in the “plant hormone signal transduction pathway”; **(B)** heatmap shows the expression of DEGs correlated with different hormones, such as auxin, cytokinin, gibberellin, abscisic acid, ethylene, brassinosteroid, jasmonic acid, and salicylic acid in “Huoju” and “Ninghaibai.” The bar represents the expression level of each gene as indicated by blue (lower expression) and dark yellow (higher expression). **(C)** Changes in the level of plant hormones, including IAA, ABA, GA3, and zeatin, under cold stress in rachises of loquat. Different letters indicate the significance at *p* < 0.05, out of which a, b, and c indicate the significant difference of “Huoju,” whereas a!, b!, and c! indicate the significant difference of “Ninghaibai.” Error bars indicate the standard deviation based on three replicates.

According to KEGG pathway analysis, the most DEG-enriched pathway was plant hormone signal transduction (Figure 4B). Plant hormones, such as auxin (IAA), cytokinin (CK), gibberellin (GA), and abscisic acid (ABA), play a vital role in modulating plant responses to abiotic stresses. Therefore, the expression patterns of DEGs related to plant hormones were further explored. A total of 101 DEGs were found to be connected with auxin (IAA)-, cytokinin (CK)-, gibberellin (GA)-, abscisic acid (ABA)-, ethylene (ET)-, brassinosteroid (BR)-, jasmonic acid (JA)-, and salicylic acid (SA)-mediated pathways (Figure 5). The expression profiles of DEGs involved in metabolism of various plant hormones are shown in Figure 5B. These DEGs exhibited distinct expression patterns in both “Huoju” and “Ninghaibai”, revealing a complicated mechanism involving phytohormones under cold stress. Among numerous hormones, there were more DEGs enriched in auxin (16 DEGs), brassinosteroid (17 DEGs), and gibberellin (16 DEGs) than in other hormones. Moreover, most of DEGs associated with phytohormones were upregulated after cold stress, and the low-temperature signal could induce an increase in the expression level of genes related to phytohormones protecting against cold injury and rendering tolerance to cold stress in loquat. The levels of IAA, ABA, GA3, and zeatin all presented a significant increase in rachis of “Huoju” under cold stress, in which the higher plant hormone content contributes to enhancing cold resistance of loquat. In the rachises of “Ninghaibai”, the levels of IAA and zeatin increased under cold stress, whereas the ABA and GA3 levels exhibited a reverse trend and sharply decreased under cold stress (Figure 5C). The higher contents of IAA, GA3, and zeatin in “Huoju” than that in “Ninghaibai” under cold stress were consistent with the expression pattern of related DEGs, in which the relative expression level of most DEGs related to plant hormones synthesis was higher in rachises of “Huoju” than that of “Ninghaibai”, suggesting that higher content of plant hormones and level of related genes may facilitate cold resistance of loquat.

### Exploration of Differentially Expressed Transcription Factors and Genes in Response to Low-Temperature Conditioning

**Figure 6:**
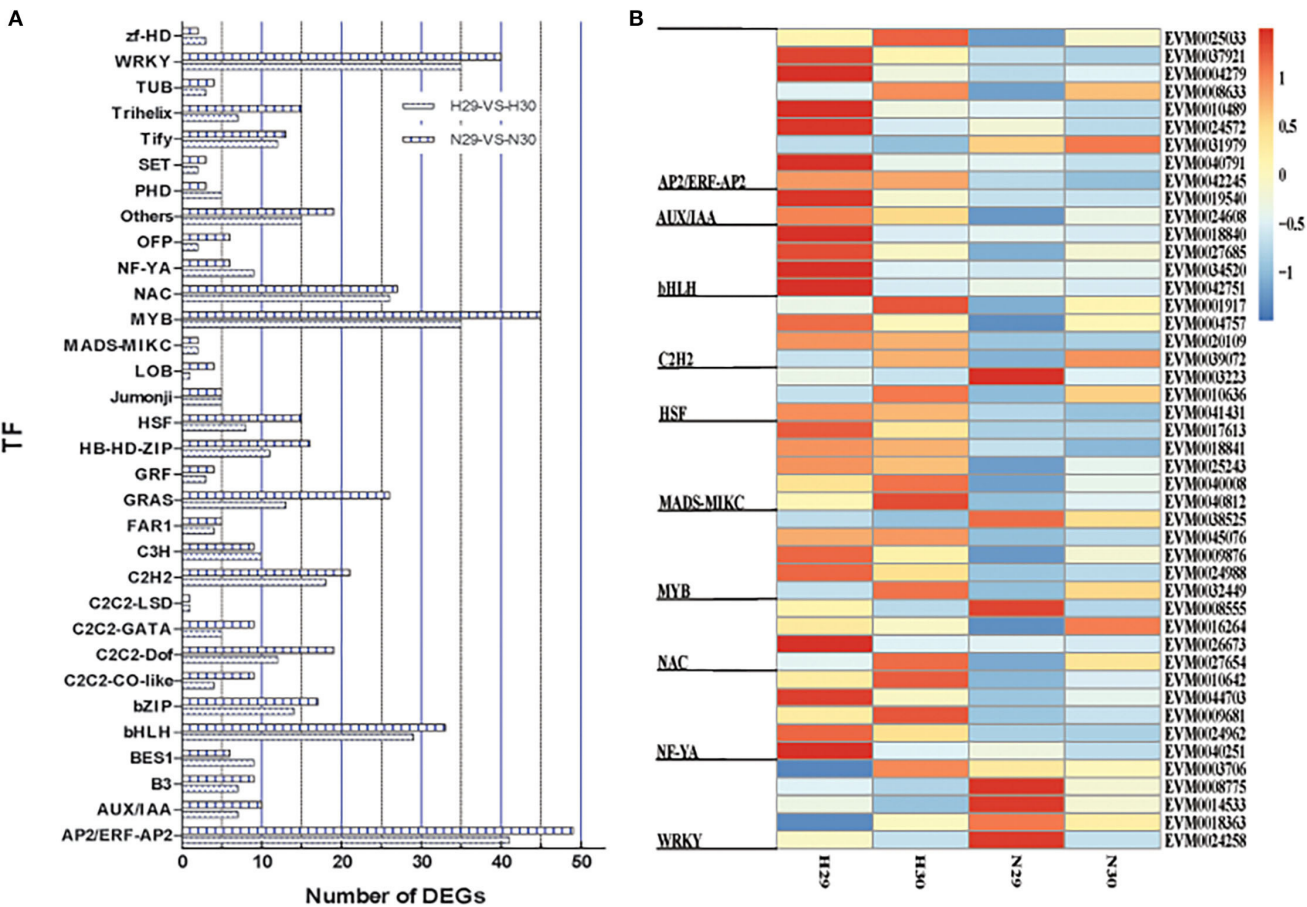
Distribution and expression profile of differentially expressed transcription factors (DETFs) under cold stress both in two loquat varieties. **(A)** The distribution of TFs; **(B)** the expression profile of TFs.

Transcription factors play the vital roles in modulating cold resistance, as reported in model plants, woody trees, and crops. We screened our assembled transcripts and predicted a total of 810 TFs from 32 families, of which 358 and 452 DETFs were in “Huoju” and “Ninghaibai”, respectively. Of these, the AP2/ERF-AP2 family contained the most DEGs (90), followed by the MYB, WRKY, bHLH, and bZIP families, with 80, 75, 62, and 31 DEGs, respectively. The C2C2-LSD family possessed the least DEGs (2). The comparative distribution of DETFs in the two loquat varieties showed that more DETFs were in “Ninghaibai” as compared to in “Huoju”, except zf-HD, PHD, NF-YA, C3H, and BES1 families (Figure 6A; Supplementary Table S5). The profiles of DETFs expression showed that there were significant differences in TFs expression between the two loquat varieties (Figure 6B; Supplementary Table S6).

**Figure 7:**
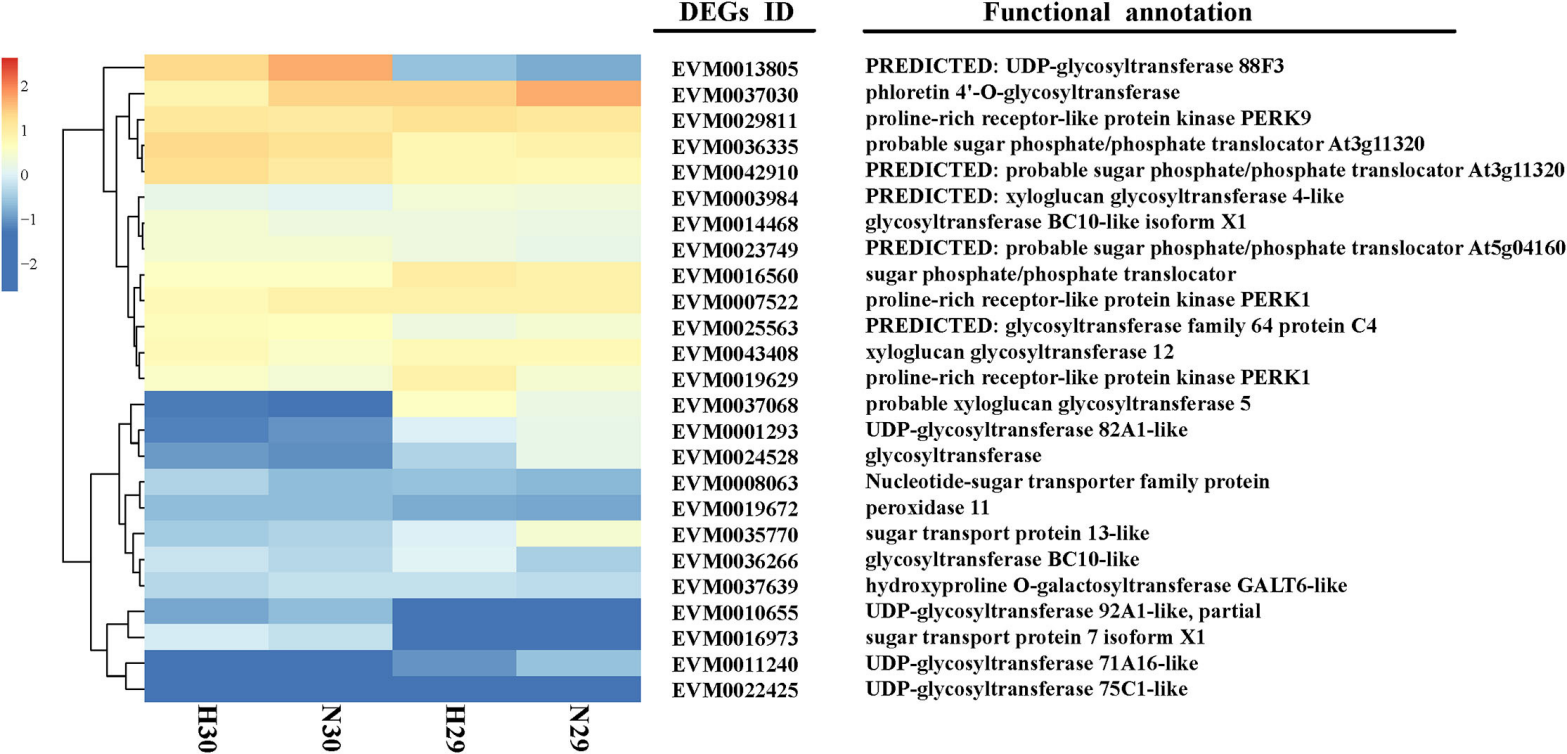
Heatmap and functional annotation of 25 DEGs related to cold resistance indicators.

The level of cold tolerance indicators was closely related to the cold resistance of varieties, and the expression profile of DEGs related to cold resistance indicators reflected the strength of cold tolerance in different loquat varieties. For this purpose, the DEGs were selected carefully following certain criteria: (1) they should be closely related to cold resistance indicators and (2) they should be highly upregulated or downregulated with at least a 3-fold difference between the sensitive and tolerant varieties. Based on the FPKM and GO or KEGG annotation analyses, we identified 21 DEGs related to sugar transport and glycosyltransferase, 3 DEGs related to proline signaling pathways, and 1 DEG associated with the POD signaling pathways, respectively (Figure 7; Supplementary Table S7).

### Phylogenetic Tree and Co-Expression Network Between DETFs and DEGs Related to Cold Resistance

**Figure 8:**
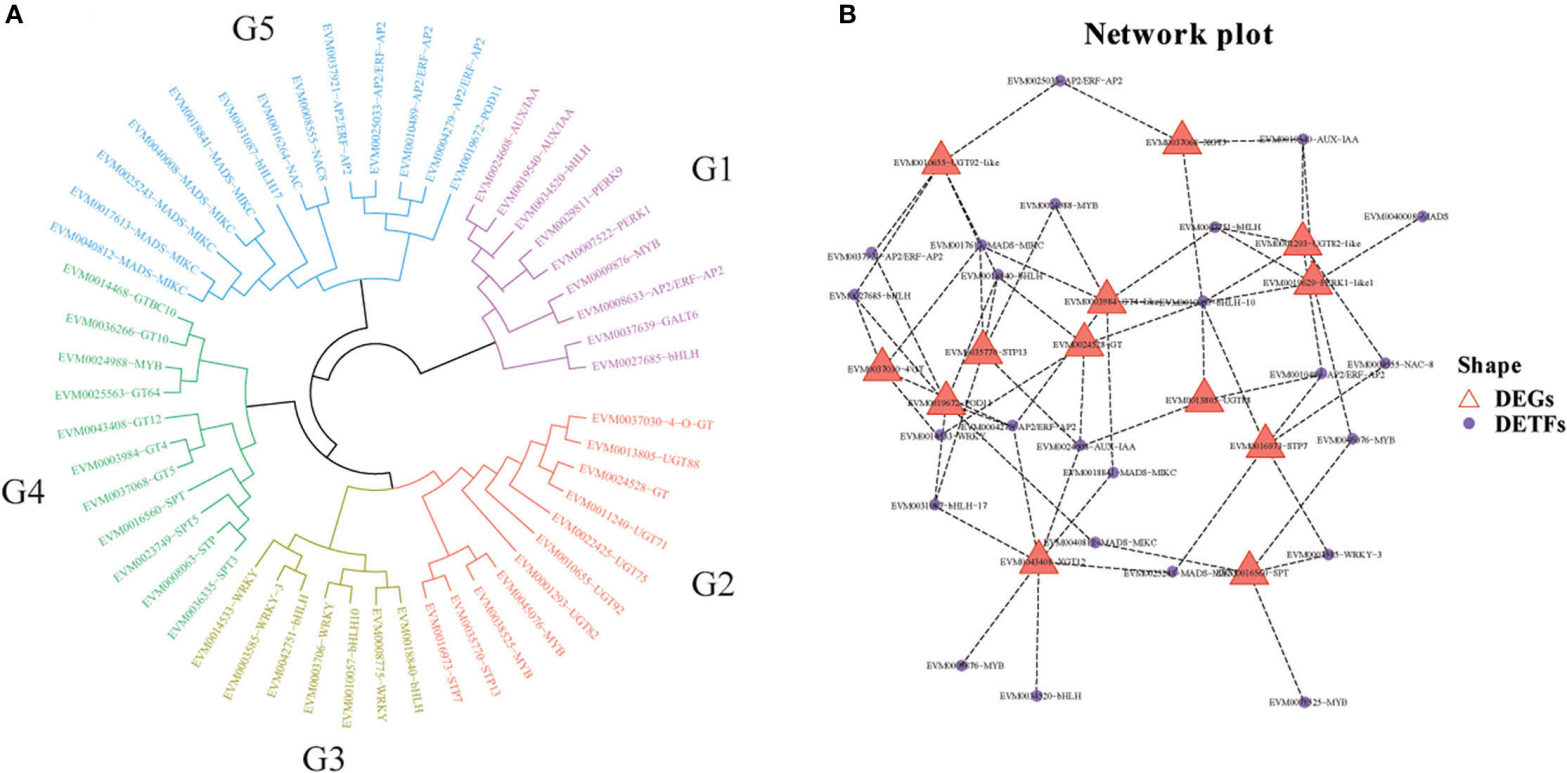
Phylogenetic tree and co-expression networks between candidates differentially expressed transcription factors (DETFs) and genes (DEGs) under cold stress. **(A)** Phylogenetic tree of DEGs and DETFs; **(B)** co-expression network between DEGs and DETFs.

To evaluate the phylogenetic relationship of DETFs and DEGs related to cold resistance in loquat, 51 full-length deduced amino acid (28 DETFs and 23 DEGs) sequences were used to construct phylogenetic tree. The results indicated that these genes were divided into five subfamilies (G1–G5) as shown in Figure 8A. Subfamilies G1, G3, and G5 were mainly composed of DETFs, including AUX/IAA, bHLH, WRKY, MADS-MIKC, NAC, and AP2/ERF-AP2. DEGs related to UDP-glycosyltransferase, glycosyltransferase, sugar phosphate/phosphate translocator, and sugar transport protein primarily concentrated upon subfamilies G2 and G4. Using the gene expression profile data, we established co-expression networks between different TFs and genes, which have been screened to be analyzed above, based on the Pearson product-moment correlation coefficient. The correlation coefficient was calculated using the expression data under cold stress. The resulting co-expression networks indicate that different TFs connected distinct genes, and there were few correlations among them (Figure 8B). Moreover, different genes or TFs could share the same/distinct TFs or genes separately. EVM0019672 (POD11), EVM0043408 (XGT12), and EVM0003984 (GT4-like) connected more TFs than other DEGs, indicating that the expression level of these genes might be jointly regulated by abundant TFs. Our DEG analysis showed that the expression of *POD11, XGT12*, and *GT4-like* was highly upregulated after cold stress in both “Huoju” and “Ninghaibai”. Meanwhile, the TFs (*bHLH, MYB*, and *MADS-MIKC*), which were closely connected to the above genes, showed similar expression trends. These TFs, including EVM0040008 (*MADS*), EVM0034520 (*bHLH*), and EVM0038525 (*MYB*), were merely connected to *PERK1-like1, XGT12*, and *SPT*, respectively, whereas other TFs connected at least two DEGs.

### Validation in DEGs and DETFs Related to Cold Tolerance With qRT-PCR

**Figure 9:**
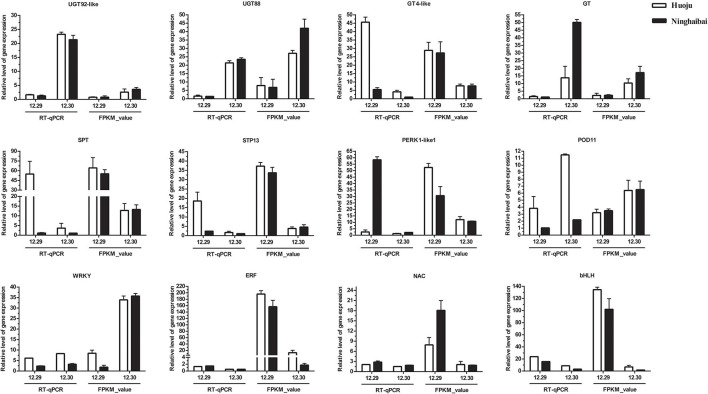
Quantitative RT-PCR validation of differential gene expression under cold stress, the transcript level of DEGs and DETFs from RT-qPCR and RNA-seq at sampling dates (12.29 and 12.30). Error bars represent the standard deviation based on three replicates.

Based on the results of the exploration of DEGs related to cold tolerance and co-expression network, 17 genes, which encoded UDP-glycosyltransferase, sugar transport protein, proline rich receptor protein, and peroxidise, were screened, and the expression of these genes differed vastly both in “Huoju” and “Ninghaibai”. Meanwhile, 4 DETFs closely associated with above 17 DEGs in phylogenetic tree and co-expression network were filtrated as vital candidate TFs that might play important roles in enhancing loquat cold resistance. To confirm the dependability of the cold-responsive gene expression profiles for DEGs and DETFs, 17 genes and 4 TFs were validated by real-time quantitative RT-PCR using gene-specific primers (Supplementary Table S1). Based on the qRT-PCR results, 8 of 17 candidate genes, including *UGT92-like* (EVM0010655), *UGT88* (EVM0013805), *GT4-like* (EVM0003984), *GT* (EVM0024528), *SPT* (EVM0016560), *STP13* (EVM0035770), *PERK1-like1* (EVM0019629), and *POD11* (EVM0019672), and 4 TFs (*WRKY, ERF, NAC*, and *bHLH*), were consistent with the gene expression levels obtained from RNA-seq data (Figure 9). After cold stress, the gene expression levels of *UGT92-like, UGT88, GT, POD11*, and *WRKY* were pronouncedly upregulated in rachises of loquat on 30 December compared with those on 29 December. On the other hand, *GT4-like, SPT, STP13, PERK1-like1, ERF, NAC*, and *bHLH* exhibited opposite dynamic trends. Moreover, there were similar change trends in the expression patterns of DEGs and DETFs, and these genes were analyzed in the co-expression network. In general, there was a potential regulatory relationship among these DEGs and DETFs responses to cold stress.

**Figure 10:**
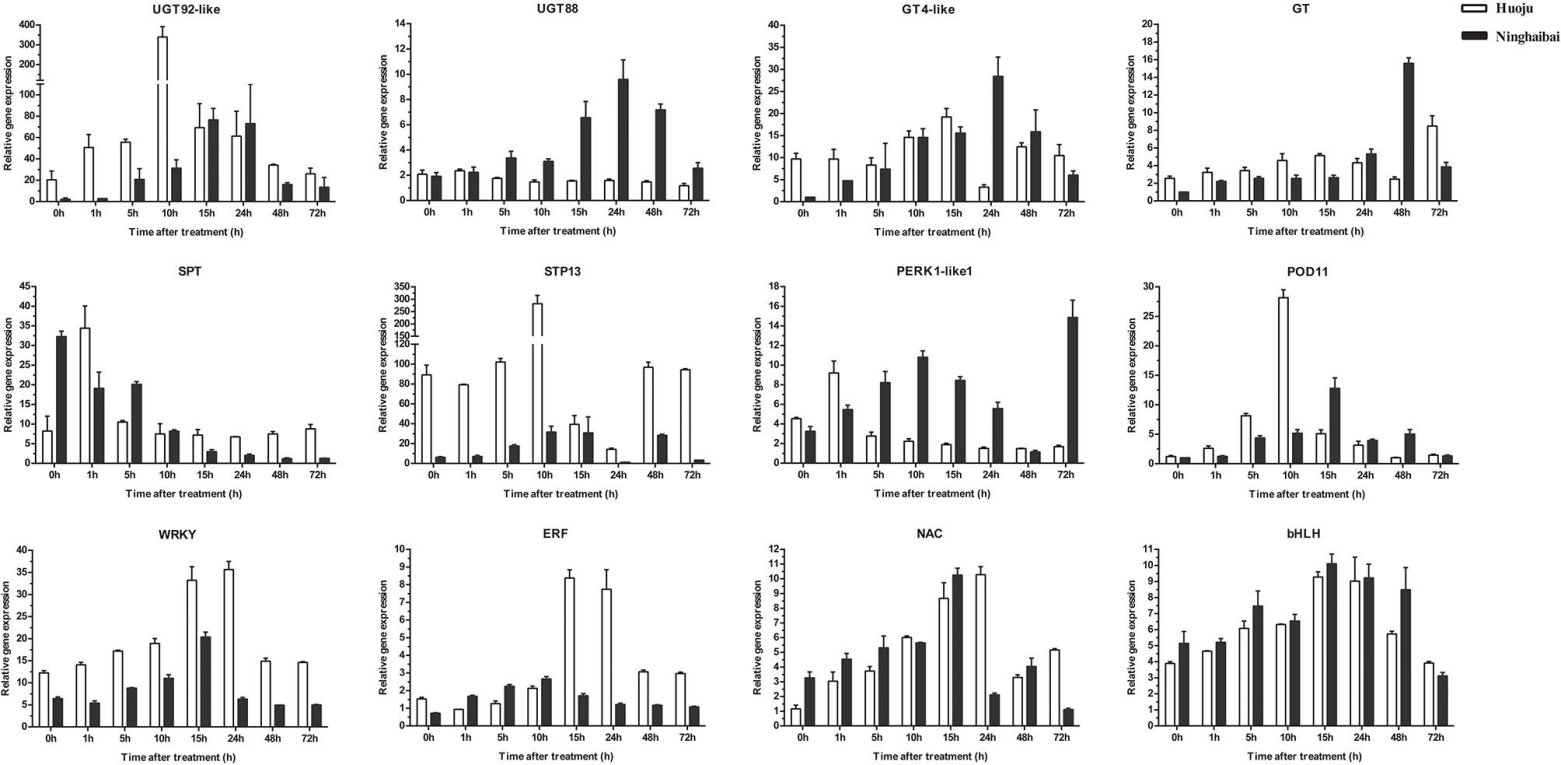
Time-course expression patterns analysis of DEGs and DETFs under low-temperature conditioning (4°C). Error bars represent the standard deviation based on three replicates.

During the low-temperature treatment at different time points, the expression patterns of most genes presented the same trend of increasing first and then decreasing. The transcript levels of *UGT92-like, UGT88, SPT, STP13*, and *POD11* reached their highest values at 10, 24, 1, 10, and 10 h, respectively, followed by decreased at the residual time points. The expression levels of 4 TFs were peaked collectively at 15 h (Figure 10). Moreover, a stronger induction of DEGs and DETFs transcript levels was observed in “Huoju” when compared to “Ninghaibai” (Figures 9, 10). The results of qRT-PCR showed that the expression profiles of the selected cold-related genes largely agreed with the RNA-seq data.

**Figure 11:**
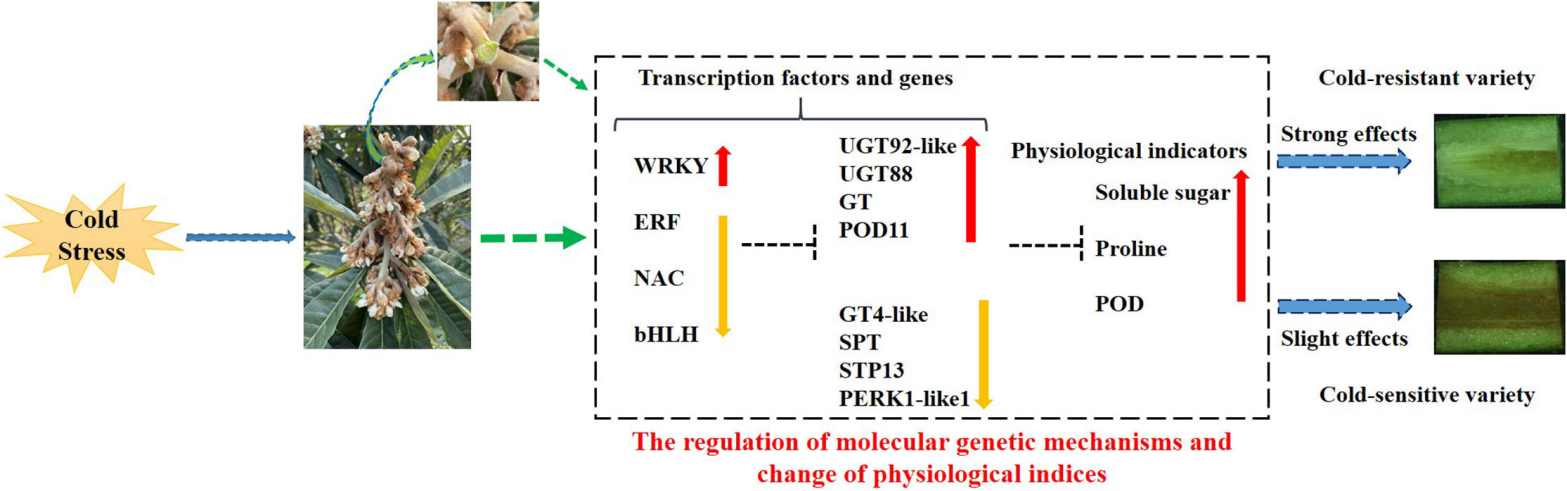
Proposed model of molecular regulatory mechanism in response to cold stress of loquat. The red up-arrow indicates a significant increase; the yellow down-arrow represents a significant reduction; the dotted line “T” indicates a potential role of positive regulation.

In summary, the different organs, especially rachises, of loquat sense low-temperature signal inducing internal changes in the expression patterns of transcription factors (TFs) and genes and the content of physiological indices to normally survive and growth under cold stress. The cold stress promoted the increase in transcript level of *WRKY*, retarded the expressions of *ERF, NAC*, and *bHLH*, and subsequently regulated the expression levels of downstream genes: *UGT92-like, UGT88, GT*, and *POD11* were upregulated; on the contrary, *GT4-like, SPT, STP13*, and *PERK1-like*1 were downregulated under cold stress. The variation in the transcript levels of TFs and genes further might affect the content of physiological indicators related to cold resistant. The improvements in soluble sugar and proline content and POD activity contributed to enhance cold resistant of loquat. The changes in gene expression levels and physiological indices exhibited stronger effects on protecting rachises of loquat from cold damage in cold-resistant variety, resulting in the lower browning rate in rachises. Inversely, in cold-sensitive variety, these changes demonstrated slighter effects on protecting against cold injury in rachises leading to higher browning rate in rachises (Figure 11).

## Discussion

Abiotic stresses can severely affect plant growth, development, crop productivity, and geographic distribution. Among abiotic stresses, cold stress is the major one that adversely limits crop productivity. Loquat was an important evergreen economic fruit tree, but it usually suffered cold stress and even freezing injury resulting in severe yield losses in winter and early spring (Lou et al., 2018). An elevation of cold resistance can compromise the risk of cold stress damage and promote or stabilize production of loquat. Nevertheless, cold tolerance is an intricate biological process controlled by numerous factors. However, limited data were available regarding either physiological or genetic mechanisms that control the cold resistance ability of loquat, illustrating that the related regulatory mechanisms would accelerate cold-resistant breeding projects and enhance fruit production (Gong et al., 2015). Based on these, in this study, the physiological indices of different organs in loquat under cold stress were determined, and a comparative transcriptome analysis was performed against the rachis organs of two loquat varieties with distinct cold resistance to reveal the potential gene regulatory pathways, as well as the cross talk of genes controlling cold resistance in loquat.

It has been confirmed that soluble sugars and proline act as important osmotic regulation substances and play the vital roles in maintaining the osmotic balance of the membrane system in plants under cold stress, and higher proline and soluble sugar contents were associated with stronger cold resistance of loquat (Sun et al., 2011; Shao et al., 2013). In this work, soluble sugar under cold stress increased obviously in rachis of cold-resistant cv. “Huoju” but decreased gradually in rachis of cold-sensitive cv. “Ninghaibai” (Figure 2A). Furthermore, the proline content under cold stress in rachis of “Huoju” was significantly higher than that of “Ninghaibai” (Figure 2B). These results indicated that cold stress significantly influenced dynamic varies of the soluble sugar and proline content in rachis of loquat. For other vital physiological indicators related to cold resistance, MDA is one of the intermediate products of lipid peroxidation and affects membrane structure, disturbing normal physiological metabolism, and the relative change in MDA content can be used as a biomarker to reflect the extent of damage to the cell membrane, as well as the ability to resist cold stress (Valitova et al., 2019). In this study, the MDA content decreased sharply under cold stress in most organs of cold-resistant cv. “Huoju”, but a lower MDA content was detected only in rachis of cold-sensitive cv. “Ninghaibai” (Figure 2C). This result was in agreement with our previous report (Zhang et al., 2021b), indicating that MDA change under cold stress could be a useful indicator for loquat cold resistance. Numerous studies have proven that cold stress-induced excess production of ROS may contribute to the development of chilling injury (Lu et al., 2019) and H_2_O_2_ and ${\text{O}}_{2}^{-}$ are major ROS types with highly reactive and toxic molecules that cause damage to protein, lipids, and nucleic acids (Gill and Tuteja, 2010). To relieve oxidative stress generated through ROS, plants evolved a valid antioxidant defense system in which antioxidant enzymes may play important roles in scavenging ROS (Flores et al., 2021). Among these antioxidant enzymes, POD, SOD, and CAT play key roles in reactive oxygen species removal, enhancing the antioxidant capacity under cold stress (Talanova et al., 2018). In the present work, the activities of POD, SOD, and CAT were mostly higher in cold-resistant cv. “Huoju” than that of cold-sensitive cv. “Ninghaibai” in test organs and the most organs of “Huoju” possessed higher antioxidant enzymes activity, accumulated less ROS, and displayed better tolerance to cold stress, whereas lower enzyme activity and more ROS were detected in “Ninghaibai” under cold stress, especially in rachis. The results obtained here were mainly consistent with that of previous work reported by Sun et al. (2011) and Zheng et al. (2015).

Previous studies have indicated that the activities of cell antioxidant enzymes, such as SOD, POD, and CAT and the contents of proline, soluble sugar, and MDA were the important physiological indexes for evaluating plant cold resistance. Meanwhile, it was feasible to assess the cold tolerance of different plants or organs using subordinate function method with multiple physiological and biochemical indexes, which could reveal the difference of respond to cold stress in different varieties or organs in grape (Gao et al., 2014), rice (Azeem et al., 2016), potato (Gupta, 2017), sugarcane (Zhang et al., 2015), and loquat (Zhang et al., 2021b), etc. In this study, the evaluation of respond to cold stress of different organs was performed using subordinate function method combined with multiple physiological indexes in two loquat varieties with difference in cold tolerance. In both two loquat varieties, the mean membership function of different physiological indicators was highest in rachis than other tested organs, which was consistent with that of previous study elaborated by Zhang et al. (2021b). In the whole, these results indicated that rachis was screened as a representative organ directly evaluated cold resistance of loquat, whereas how rachis perceived cold signal, regulated the synthesis of soluble sugar and proline, and thus enhanced loquat cold resistance, and the related physiological and biochemical mechanisms need to be further studied in depth.

The transcriptome is the complete set and quantity of transcripts in a tissue at a specific developmental stage or under physiological conditions. Based on transcriptomic information, an understanding of abundant biological processes is now possible, for example, gene expression profiles under experimental treatment or infection, gene regulation and discovery, functional annotation of genes, and exploration of tissue biomarkers, among others (Raza et al., 2021). Therefore, transcriptome analysis is an effective tool to study gene functions or regulatory networks participating in the responding of cold stress. This approach identified DEGs or DETFs that played crucial roles in protecting against cold injury in pear (Li et al., 2016), rice (Pradhan et al., 2019), apple (Wang et al., 2016), and wheat (Naydenov et al., 2010). Certain genes related to cold stress, such as *EjICE1* (Hong et al., 2018), *EjLGA1* (Wu et al., 2018a), *EjDHN1* (Xu et al., 2018a), *EjbHLH1*, and *EjMYB2* (Xu et al., 2019), have been identified in loquat. However, up to date, less is known about the regulatory mechanisms of cold tolerance in loquat. To excavate the genes related to cold resistance and illustrate their transcriptional regulatory network during cold stress in loquat, we performed a comparative transcriptome analysis of rachises on the cold-resistant variety “Huoju” and cold-sensitive variety “Ninghaibai”. We obtained 21.69–24.16 M clean reads with Q30 bases from “Huoju” and “Ninghaibai”, and a total of 7,860 DEGs were functionally annotated by several complementary approaches. The annotations provided a crucial resource for surveying specific functions, biological processes, and metabolic pathways for researching the cold-responsive mechanism of loquat. The results of KEGG pathway enrichment analysis indicate that the plant hormone signal transduction was the top pathway and that DEGs were mainly enriched in this pathway. Plant hormones are involved in diversified physiological processes in plants, including metabolism, morphogenesis, and growth (Pandey et al., 2021), and the plant hormone functions as vital regulators under cold stress have been studied (Kumar et al., 2008; Xue et al., 2010; Eremina et al., 2016). IAA, ABA, GA3, and zeatin, as major plant endogenous hormones types, played the important roles in coping with stress and ensuring the yield of economic crops and the increase in the level of plant hormones could enhance resistance to cold stress in rice (Sun et al., 2022), tomato (Imen et al., 2018), cucumber (Huang et al., 2020), and pear (Yang and Huang, 2018), etc. There were higher plant hormone content and related gene expression level in “Huoju” compared with “Ninghaibai” and it might endow “Huoju” with stronger cold resistance. These results indicated that plant hormones and related genes might play a vital role in cold tolerance of loquat. Therefore, plant hormone signal transduction and DEGs closely related to this pathway will be the key research objects for further study.

Soluble sugars, proline, and POD have been identified as vital physiological metabolites involved in loquat cold resistance under low-temperature conditioning. The genes or TFs that mediated the biosynthesis, transport, signal transduction, and distribution of these metabolic pathways were closely related to cold tolerance. *CsSWEET16*, as a member of the sugar transporter family, plays an important role in various biological processes, including responses to cold stress in tea (Wang et al., 2018). Zhao et al. (2020) demonstrated that glycosyltransferase *CsUGT78A15* could be induced by cold stress and contribute to the formation of eugenol glucoside and function in promoting cold tolerance in *Camellia sinensis* under low-temperature conditions. Several proline-rich proteins have been evaluated in *Arabidopsis* and *Poncirus trifoliata*. Overexpression of *CcHyPRP* (Priyanka et al., 2010) and *PtrPRP* (Peng et al., 2015) leads to enhanced cold resistance by reducing electrolyte leakage and MDA content to maintain membrane integrity and ROS homeostasis. POD, as one of the important antioxidant enzymes, scavenges or regulates ROS levels to protect against oxidative injury, enhancing antioxidant capacity under cold stress. *RCI3* and *RCI35* encode POD that elevates cold resistance when overexpressing in plants (Llorente et al., 2010; Zhou et al., 2016). TFs serve as the regulatory factors of the promoter activity of genes to modulate gene transcript levels, improving plant tolerance to stresses. TFs, such as those in the *WRKY, ERF, NAC*, and *bHLH* families, have been reported to induce the unregulated expression of genes related to cold stress, rendering enhanced resistance to chilling or freezing temperature conditioning (Huang et al., 2015; Jin et al., 2017; Xu et al., 2019). In this study, some cold-induced DEGs related to sugar, glycosyltransferase, proline, peroxidase, and some TFs were identified from transcriptome data. The results of co-expression network analysis indicated that there was a potential regulatory relationship between DEGs and DETFs. Moreover, the RNA-seq data revealed that the expression pattern of genes varied significantly between “Huoju” and “Ninghaibai”. The qRT-PCR results showed that the expression levels of these DEGs, which encodes UGT, GT, SPT, STP, PERK, and POD, and DETFs were highly consistent with the RNA-seq results. Moreover, there was a stronger induction of gene transcript levels in the cold-resistant genotype “Huoju” when compared to cold-sensitive genotype “Ninghaibai”. These results indicated UGT, GT, SPT, STP, PERK, and POD and DETFs were induced quickly and enhanced cold resistance and might play a potential role in enhancing loquat cold tolerance. Therefore, the functional characteristics and molecular mechanisms of these genes in the control of loquat cold resistance remain to be elucidated.

## Conclusion

In the present work, the rachis was screened as an important organ to evaluate the cold resistance of loquat under low-temperature conditioning. Totally, 3,513 and 4,347 DEGs involved in multiple regulatory pathways of cold stress were, respectively, identified *via* RNA-seq analysis against the rachis samples collected from the cold-resistant cv. “Huoju” and the cold-sensitive cv. “Ninghaibai”. Plant hormone signal transduction was the top pathway that enriched most DEGs. A total of eight DEGs related to sugar, glycosyltransferase, proline, and POD and four DETFs (*WRKY, ERF, NAC*, and *bHLH*) could be more strongly induced in “Huoju” than in “Ninghaibai”, suggesting that these DEGs might play the vital roles in enhancing cold resistance in loquat. The results presented here offer new insights into the molecular regulation mechanism underlying the cold stress response in loquat. Furthermore, the data obtained in this work could be used as an empirical reference for identifying the candidate genes that hold great potential for genetic engineering and for the breeding process of novel germplasms with reinforced cold resistance.

## Data Availability Statement

The original contributions presented in the study are publicly available. This data can be found here: NCBI, PRJNA835244.

## Author Contributions

XZ and HA organized the entire project and edited the manuscript. JZ and HA performed the experiments and data analysis. FX and BZ help to review and modify the manuscript. All authors read and approved the final manuscript.

## Funding

This work was supported by the Agriculture Applied Technology Development Program of the Shanghai Agriculture Committee (No. 2018-1-7) and Basic Research Project of SAAS (JCYJ222201).

## Conflict of Interest

The authors declare that the research was conducted in the absence of any commercial or financial relationships that could be construed as a potential conflict of interest.

## Publisher's Note

All claims expressed in this article are solely those of the authors and do not necessarily represent those of their affiliated organizations, or those of the publisher, the editors and the reviewers. Any product that may be evaluated in this article, or claim that may be made by its manufacturer, is not guaranteed or endorsed by the publisher.

## Supplementary Material

Supplementary Figure S1 Difference in the expression of genes in rachis of loquat and Venn diagram showing numbers of upregulated and downregulated DEGs in each sampling period. Arabic numbers in center indicate the DEGs common to two libraries; Arabic numbers in margin indicate the DEGs individually owned in each library, respectively.

Supplementary Figure S2 Functional annotation of loquat transcripts based on the clusters of orthologous (COG) groups dataset. A total of 2,932 DEGs (Huoju: 1337, Ninghaibai: 1595) were annotated and classified into 26 categories. X-axis indicated different functional classifications and y-axis indicated the number of DEGs in each COG class.

## Abbreviations

GO: Gene Ontology
COG: Clusters of Orthologous Groups
KEGG: Kyoto Encyclopedia of Genes and Genomes
DEGs: differentially expressed genes
DETFs: differentially expressed transcriptional factors
qRT-PCR: real-time quantitative reverse transcription-polymerase chain reaction
MDA: malondialdehyde
SOD: superoxide dismutase
POD: peroxidase
CAT: catalase
NR: nonredundant
NT: nucleotide
NCBI: national center for biotechnology information
FPKM: fragments per kilobase per million mapped reads.
