# Supplementary figures and images for: Transcriptomic Analysis Reveals Potential Gene Regulatory Networks Under Cold Stress of Loquat (Eriobotrya japonica Lindl.)
Source: Front Plant Sci. 2022 Jul 22;13:944269. doi: 10.3389/fpls.2022.944269 (PMC9354853; doi:10.3389/fpls.2022.944269)

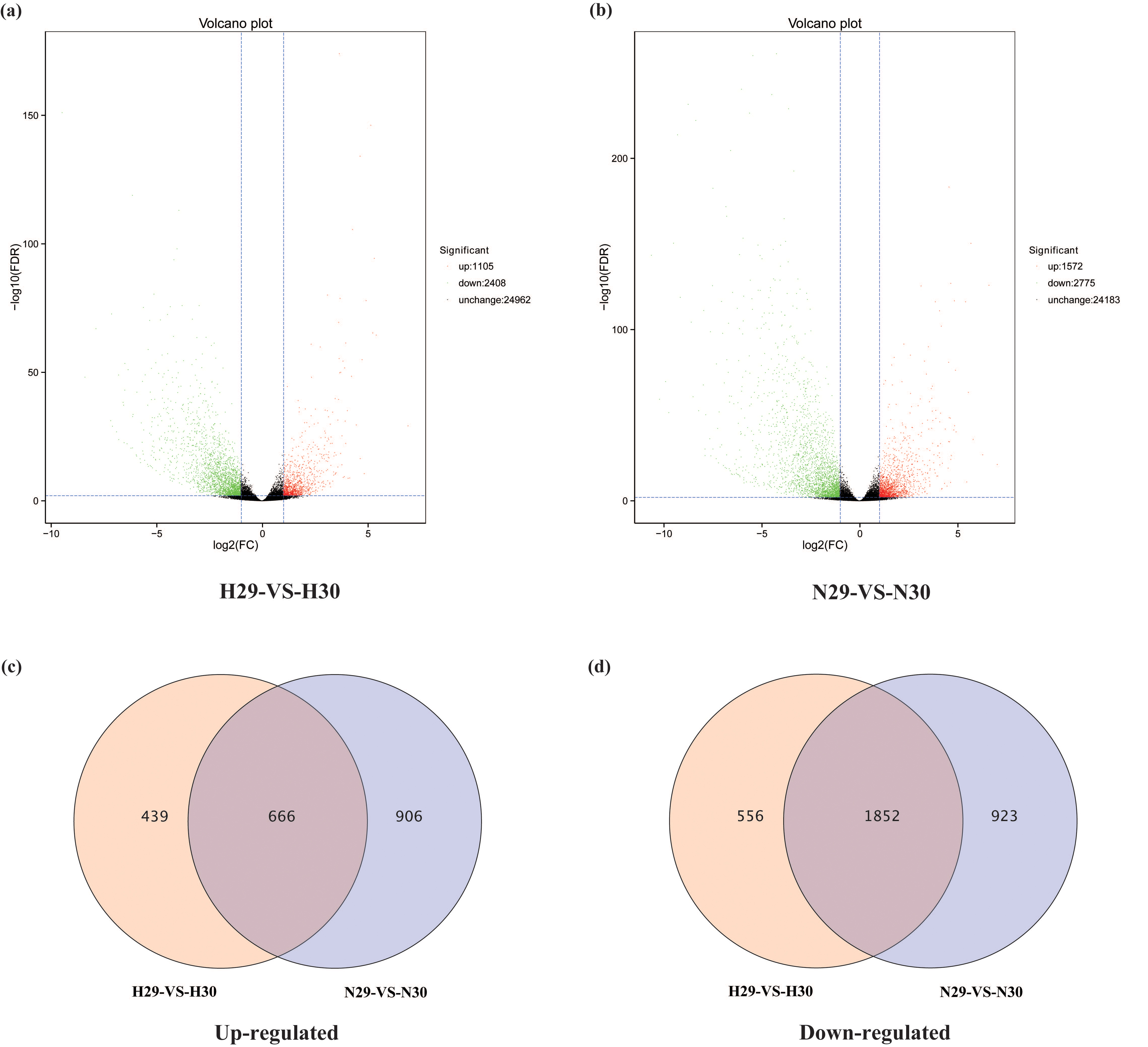

Supplement: Supplementary Figure S1 — Difference in the expression of genes in rachis of loquat and Venn diagram showing numbers of upregulated and downregulated DEGs in each sampling period. Arabic numbers in center indicate the DEGs common to two libraries; Arabic numbers in margin indicate the DEGs individually owned in each library, respectively. [file Image_1.JPEG]

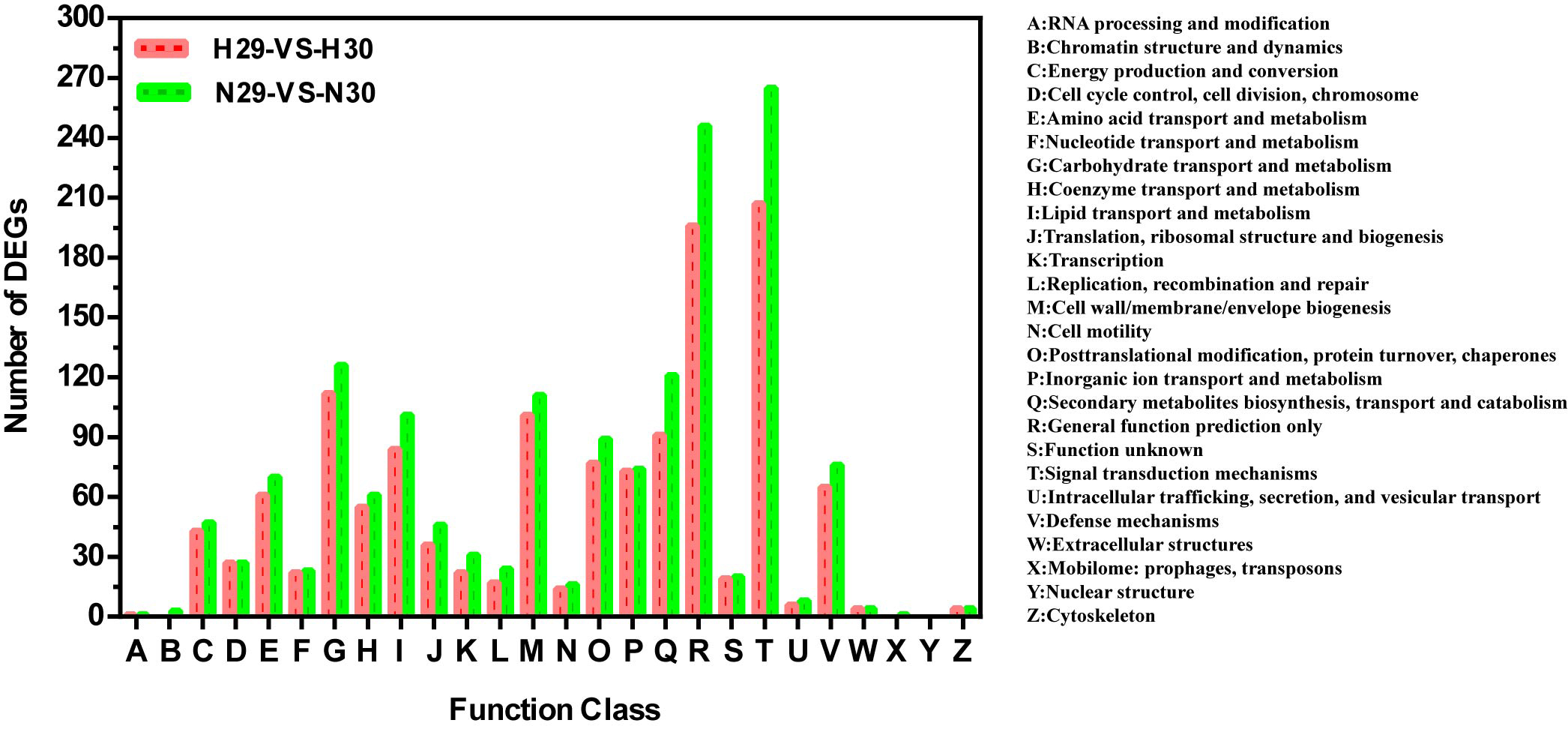

Supplement: Supplementary Figure S2 — Functional annotation of loquat transcripts based on the clusters of orthologous (COG) groups dataset. A total of 2,932 DEGs (Huoju: 1337, Ninghaibai: 1595) were annotated and classified into 26 categories. X-axis indicated different functional classifications and y-axis indicated the number of DEGs in each COG class. [file Image_2.JPEG]
